# Unraveling resistance to immune checkpoint inhibitors in HNSCC: from mechanisms to combination therapies

**DOI:** 10.20517/cdr.2025.153

**Published:** 2025-12-05

**Authors:** Wenchao Zhao, Qingqing Luo, Bowen Yuan, Huaxin Duan, Siqing Jiang

**Affiliations:** ^1^Department of Oncology, Hunan Provincial People’s Hospital, the First Affiliated Hospital of Hunan Normal University, Changsha 410000, Hunan, China.; ^2^Key Laboratory of Study and Discovery of Small Targeted Molecules of Hunan Province, Changsha 410000, Hunan, China.; ^3^Hunan Hepatobiliary and Pancreatic Cancer Clinical Medical Research Center, Changsha 410000, Hunan, China.; ^4^Department of Pathology, the Third Xiangya Hospital of Central South University, Changsha 410013, Hunan, China.; ^5^Department of Comprehensive Chemotherapy/Head and Neck Cancer, Hunan Cancer Hospital, the Affiliated Cancer Hospital of Xiangya School of Medicine, Central South University, Changsha 410013, Hunan, China.

**Keywords:** R/M HNSCC, immunotherapy, therapy resistance, PD-1, therapeutic strategies

## Abstract

Head and neck squamous cell carcinoma (HNSCC), which arises from the mucosal linings of the oral cavity, pharynx, and larynx, represents the most prevalent head and neck malignancy. This cancer is notable for its elevated incidence and substantial mortality. The intricate anatomy of the region contributes to marked tumor heterogeneity, rendering the pursuit of effective therapeutic regimens a crucial aspect of enhancing clinical outcomes. Recently, the advent of immune checkpoint blockade, particularly agents targeting programmed death-1 (PD-1) and cytotoxic T-lymphocyte-associated protein 4, has introduced significant advancements within the oncological landscape, including for HNSCC. The introduction of immune checkpoint inhibitors, specifically the PD-1 blockers pembrolizumab and nivolumab, has established a new therapeutic standard for recurrent/metastatic HNSCC (R/M HNSCC). However, the clinical benefit is not universal, as a primary challenge remains the high incidence of treatment resistance. Consequently, a majority of patients (approximately 60%-70%) with R/M HNSCC derive minimal or no benefit from this form of immunotherapy, highlighting the critical need to understand the underlying resistance mechanisms. This review comprehensively discusses the types of immunotherapy resistance in HNSCC and the underlying mechanisms contributing to resistance. Furthermore, it reviews current strategies to overcome immunotherapy resistance, providing new perspectives for improving therapeutic efficacy in HNSCC.

## INTRODUCTION

Globally, cancers of the head and neck impose a significant burden, being ranked as the seventh most common malignancy. Epidemiological studies from 2020 recorded around 890,000 new diagnoses and 450,000 deaths attributable to this disease group^[1]^. A concerning upward trend in incidence has been observed, and forecasts indicate a potential 30% increase by the end of this decade, culminating in roughly 1.08 million new cases per year by 2030^[2]^. Tobacco smoking is a major risk factor for head and neck squamous cell carcinoma (HNSCC), significantly increasing disease susceptibility. Additionally, alcohol consumption, environmental pollutant exposure, and viral infections - specifically human papillomavirus (HPV) and Epstein-Barr virus (EBV) - are recognized as high-risk factors for HNSCC development. HPV and EBV infections are known etiological contributors to oropharyngeal cancer and nasopharyngeal cancer, respectively^[3]^.

The programmed death-1 (PD-1)/programmed death-ligand 1 (PD-L1) axis has emerged as a cornerstone for immunotherapy in HNSCC. Agents targeting this pathway, such as pembrolizumab and nivolumab, have established clinical efficacy, leading to their regulatory approval. The current first-line standard of care for recurrent/metastatic HNSCC (R/M HNSCC) disease often involves a combination of immune checkpoint inhibitors (ICIs) with platinum. Beyond this established setting, the therapeutic landscape is expanding, with ongoing clinical investigations exploring ICIs concurrently with chemoradiotherapy for locally advanced HNSCC and as a neoadjuvant approach prior to surgical resection^[4]^. Despite significant advancements in immunotherapy for R/M HNSCC, overall survival (OS) rates remain limited. The data show that immunotherapy has improved the 5-year survival rate from 5.0% to 15.4%-23.9%; however, approximately 60% of patients eventually develop resistance, and only one-third of patients achieve long-term disease control^[5]^.

This review provides a comprehensive focus on resistance mechanisms, offers an integrated framework for classifying and addressing different types of resistance, and provides updated, specific and actionable strategies for overcoming resistance in clinical practice in HNSCC.

## CURRENT STATUS OF IMMUNOTHERAPY FOR HNSCC

ICIs, such as anti-PD-1 monoclonal antibodies (mAbs), have become the standard first-line treatment for R/M HNSCC and are recommended by multiple guidelines^[6-8]^. Among the clinically approved anti-PD-1 immunotherapies are nivolumab, pembrolizumab, and toripalimab, all of which are humanized immunoglobulin G4 (IgG4) mAbs.

Their primary mechanism involves blocking the engagement of PD-1 with its ligands (PD-L1/PD-L2), thereby preventing the negative regulation of T-cell activity. This intervention reinvigorates tumor-specific T cells, promoting their activation, proliferation, and cytotoxic capabilities against tumor cells. The restored anti-tumor immunity is largely mediated through the reactivation of cluster of differentiation 8 positive (CD8^+^) cytotoxic T lymphocytes (CTLs) and the enhanced phagocytic function of antigen-presenting cells (APCs)^[9]^. In addition to the approved anti-PD-1 mAbs, PD-L1 checkpoint inhibitors primarily bind to the programmed cell death ligand on the surface of tumor cells. Here, we present some key clinical trials of immunotherapy in R/M HNSCC^[5,10-17]^ [Table 1].

**Table 1:** Key clinical trials of immunotherapy in R/M HNSCC

**Trial name**	**Phase**	**Population**	**Treatment Arms**	**ORR (%)**	**Median OS (months)**	**Grade 3/4 TRAEs (%)**	**Key findings**
KEYNOTE-012^[10]^	Ib	R/M HNSCC	Pembrolizumab	18	8.0	9	First trial showing efficacy of anti-PD-1 in HNSCC
CheckMate-141^[18]^	III	Platinum-refractory R/M HNSCC	Nivolumab *vs.* SOC	13.3 *vs.* 5.8	7.7 *vs.* 5.1	15.3 *vs.* 36.9	FDA approval for nivolumab in second-line treatment of HNSCC
KEYNOTE-055^[12]^	II	Platinum and cetuximab refractory R/M HNSCC	Pembrolizumab	16	8	15	Pembrolizumab exhibited clinically meaningful anti-tumor activity and an acceptable safety profile
KEYNOTE-040^[19]^	III	Platinum-refractory R/M HNSCC	Pembrolizumab *vs.* SOC	22.6 *vs.* 16.7	8.4 *vs.* 6.9	13 *vs.* 35	Significant OS improvement with pembrolizumab
KEYNOTE-048^[13]^	III	R/M HNSCC	Pembrolizumab *vs.* Pembrolizumab + chemotherapy *vs.* EXTREME	Undisclosed	11.5 *vs.* 13.0 *vs.* 10.7	55 *vs.* 85 *vs.* 83	Led to 1st line approval in CPS ≥ 1 patients
CheckMate-651^[14]^	III	R/M HNSCC	Nivolumab plus ipilimumab *vs*. EXTREME	34.1 *vs.* 36.0	13.9 *vs.* 13.5	70.7 *vs.* 28.2	No significant OS improvement
KEYNOTE-412^[15]^	III	Unresected locally advanced HNSCC	Pembrolizumab plus chemoradiotherapy *vs.* placebo plus chemoradiotherapy	-	Not reached *vs.* 47.7	92 *vs.* 88	Pembrolizumab plus chemoradiotherapy did not significantly improve EFS
CheckMate-714^[16]^	II	Platinum-refractory R/M HNSCC	Nivolumab plus ipilimumab *vs.* Nivolumab	13.2 *vs.* 118.3	Undisclosed	5.7 *vs.* 3.7	No significant PFS and OS improvement
KESTREL^[17]^	III	R/M HNSCC	Durvalumab + tremelimumab *vs.* durvalumab *vs.* EXTREME	Undisclosed	10.9 *vs.* 11.2 *vs.* 10.9	8.9 *vs.* 19.1 *vs.* 53.1	No significant OS improvement

R/M HNSCC: Recurrent/metastatic head and neck squamous cell carcinoma; ORR: overall response rate; OS: overall survival; TRAEs: treatment-related adverse events; PD-1: programmed death-1; SOC: standard of care; FDA: U.S. Food and Drug Administration; CPS: combined positive score; EFS: event-free survival; PFS: progression-free survival.

Despite the transformative impact of PD-1/PD-L1 checkpoint inhibitors on the management of R/M HNSCC, a significant clinical challenge persists. The efficacy of this immunotherapeutic strategy is markedly limited by primary resistance, which prevents durable responses in a substantial majority (over 60%) of patients, underscoring the need to overcome this barrier to improve outcomes. Future trials should prioritize biomarker-driven patient stratification and explore rational combinations with radiotherapy or targeted agents to expand durable response rates.

## FACTORS PREDICTING IMMUNOTHERAPY EFFICACY

**Figure 1:**
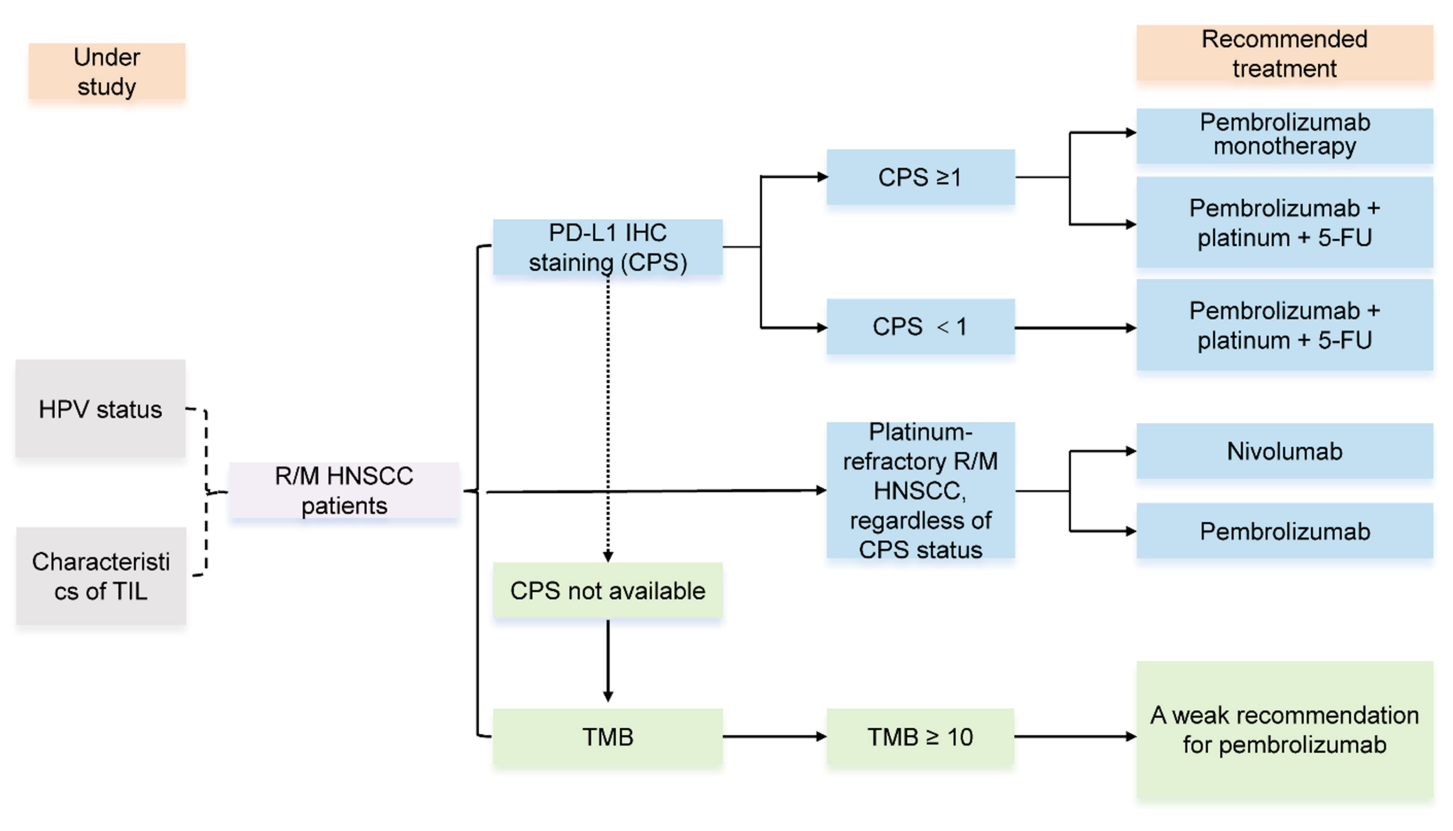
Biomarkers to evaluate the efficacy of immunotherapy. PD-L1 IHC staining and TMB validated by clinical guidelines, HPV status and characteristics of TIL are under study. PD-L1: Programmed death-ligand 1; IHC: immunohistochemistry; TMB: tumor mutation burden; HPV: human papillomavirus; TIL: tumor immune infiltration; R/M HNSCC: recurrent/metastatic head and neck squamous cell carcinoma; CPS: combined positive score; 5-FU: 5-fluorouracil.

As previously mentioned, immunotherapy is currently effective in the treatment of R/M HNSCC and is undergoing many clinical trials in both first-line and later-line settings. Currently, the expression levels of PD-L1 and tumor mutation burden (TMB) have been recommended by guidelines for evaluating the efficacy of immunotherapy in HNSCC. However, immunotherapy, guided by predictive biomarkers, is not effective for all patients. Besides, HPV status and the characteristics of tumor immune infiltration (TIL) in immunotherapy for HNSCC, based on existing clinical trial results, lack sufficient evidence and need further study [Figure 1]. Therefore, finding biomarkers that predict the efficacy of immune response treatment has become an urgent task.

### PD-L1 expression

Within the predictive biomarker landscape for immune checkpoint inhibition, the expression level of PD-L1 holds significant prominence. Its evaluation in tumor samples typically relies on two principal methodologies: tumor proportion score (TPS), which quantifies expression on tumor cells alone, and combined positive score (CPS), which provides a more comprehensive assessment by incorporating staining on both tumor and associated immune cells^[20-22]^ [Table 2]. Clinical data from various studies, including the phase II KEYNOTE-055 trial (NCT02255097), have established a general association between higher PD-L1 expression (using a CPS ≥ 1% cutoff) and improved OS (18% *vs.* 12% in CPS < 1% patients). However, the documented efficacy in some PD-L1-negative cohorts confirms that this biomarker, while informative, lacks absolute predictive power, highlighting the contribution of other resistance and response mechanisms^[12]^. Clinical guidelines now strongly endorse pembrolizumab, either as monotherapy or in combination with platinum and 5-fluorouracil (5-FU), as a first-line treatment for R/M HNSCC in patients with a CPS ≥ 1%. This recommendation is substantiated by the KEYNOTE-048 (NCT02358031) and KEYNOTE-040 (NCT02252042) trials, which demonstrated a concordant association between higher CPS scores and enhanced efficacy of immunotherapy^[13,20]^. Supporting evidence also comes from the CheckMate-141 trial (NCT02105636), which utilized the TPS method. In this trial, patients treated with nivolumab whose tumors had a TPS > 1% showed improved progression-free survival (PFS), although a significant OS benefit was not observed across all groups^[23]^. For individuals with a CPS < 1%, the recommended regimen is pembrolizumab combined with platinum and 5-FU. Meanwhile, both pembrolizumab and nivolumab remain therapeutic options for patients with platinum-refractory R/M HNSCC, irrespective of their CPS status^[24]^.

**Table 2:** Comparison of PD-L1 assessment methods in HNSCC

**Feature**	**TPS**	**CPS**
Definition	Percentage of tumor cells with PD-L1 membrane staining	Sum of PD-L1 staining tumor cells and immune cells per 100 tumor cells
Cells evaluated	Tumor cells only	Tumor cells + immune cells
Advantages	Simple, standardized	More comprehensive immune assessment
Limitations	Ignores immune cell PD-L1 expression	More complex scoring system
Clinical utility	Predictive in some cancers	Better sensitivity in HNSCC
Preferred cutoff	≥ 50%	≥ 1%

PD-L1: Programmed death-ligand 1; HNSCC: head and neck squamous cell carcinoma; TPS: tumor proportion score; CPS: combined positive score.

### TMB and microsatellite instability

The concept of TMB provides a metric for the genomic alteration frequency in cancers, defined as the number of protein-altering (nonsynonymous) mutations accumulated per megabase of the sequenced coding region. An elevated mutational load is hypothesized to increase the repertoire of tumor neoantigens presented on the cell surface. This heightened antigenic diversity promotes immunogenic recognition, thereby potentially sensitizing tumors to immunotherapies that unleash T-cell cytotoxicity, such as anti-PD-1/PD-L1 agents. The KEYNOTE-158 (NCT02628067) study evaluated the correlation between TMB and the efficacy of pembrolizumab in solid tumors, showing that pembrolizumab treatment in patients with TMB ≥ 10 mut/mb resulted in a higher overall response rate (ORR) (29% *vs.* 6%)^[25]^. Additionally, the KEYNOTE-012 (NCT01848834) study analyzed the data on TMB and pembrolizumab treatment in R/M HNSCC, finding a positive correlation between TMB and ORR. In the subgroup analysis, TMB ≥ 175 mut/exome performed better than TMB < 175 mut/exome; however, the median OS showed no significant improvement, and there was no correlation between TMB and tumor tissue CPS scores^[26]^. Therefore, American Society of Clinical Oncology (ASCO) guidelines recommend a weak recommendation for pembrolizumab in patients with high TMB and rare R/M HNSCC. When CPS is unavailable, TMB testing is recommended for patients with R/M HNSCC, with TMB ≥ 10 considered high and associated with clinical benefit from PD-1 inhibitors^[24]^. The microsatellite instability (MSI) status has been considered an important predictive factor for immunotherapy efficacy in various cancers, and it was approved by the U.S. Food and Drug Administration (FDA) in 2017 for use in MSI-high solid tumors across cancers. However, only 1%-3% of patients with HNSCC report MSI-high status, and routine testing for this in HNSCC patients is not currently recommended^[19]^.

### HPV status

Infection with high-risk HPV is an established etiological factor for a distinct subset of HNSCC. HPV-positive (HPV+) tumors constitute a distinct clinical and biological entity compared to HPV-negative (HPV-) HNSCC, characterized by divergent genetic profiles, mutational patterns, and immune phenotypes^[27]^. Nevertheless, the value of HPV status as a predictive marker for immunotherapy efficacy remains poorly defined and continues to be a focus of intensive research^[28,29]^. The immunogenic nature of HPV, driven by the persistent expression of viral oncoproteins (E6/E7), typically induces a robust anti-tumor immune response. This immunological feature is well reflected in the tumor microenvironment (TME) of HPV+ HNSCC: such TMEs are frequently characterized by a more pronounced inflammatory phenotype, including increased infiltration of CD8^+^ CTLs, cluster of differentiation 4 positive (CD4^+^) T helper 1 (Th1) cells, and APCs, as well as higher concentrations of pro-inflammatory cytokines and chemokines. Several clinical trials have investigated the correlation between HPV status and ICI responses. While early subgroup analyses from CheckMate 141 and KEYNOTE-040 suggested limited survival benefit of PD-1 inhibitors in HPV+ HNSCC^[11,19]^, subsequent evidence reveals this reflects quantitative limitations in predictive power rather than true inefficacy. HPV+ tumors exhibit marked heterogeneity: A subset with intact antigen presentation machinery and high CD8^+^ TIL density shows robust responses^[30,31]^. Thus, HPV status alone is insufficient for patient selection; it must be integrated with spatial immune mapping and genomic classifiers.

### Characteristics of TIL

In addition to PD-L1 expression, TMB, and MSI status, there have been new explorations into biomarkers for predicting the efficacy of immunotherapy in HNSCC. Similar to lung cancer and melanoma, HNSCC exhibits significant TIL. Some studies suggest that tumor lymphocytic infiltration is associated with prognosis. For instance, the density of immune cell infiltration in HNSCC has been established as a positive prognostic indicator for patients receiving ICIs. Studies indicate that immunologically “hot” tumors, which are markedly enriched with CD8^+^ T cells and TILs, orchestrate potent anti-tumor responses via the localized production of cytokines including interferon-γ (IFN-γ), perforin, and granzymes^[32]^. Complementing this, research has also highlighted the role of tumor-infiltrating natural killer (NK) cells in predicting favorable responses to immunotherapy. A key mechanism involves IFN-γ secretion by NK cells, which activates Th1 lymphocytes and subsequently contributes to the reversal of an immunosuppressive milieu, enhancing the overall efficacy of the treatment^[33]^.

In the future, validation of composite biomarkers - incorporating genomic, immunophenotypic, and microenvironmental features - is critical to better stratify patients. Future studies should also explore dynamic biomarkers and liquid biopsies to capture temporal evolution of resistance. Ultimately, advancing predictive accuracy will require multimodal algorithms that reconcile tumor intrinsic features with immune contexture, enabling more personalized and effective immunotherapy strategies in HNSCC.

## MECHANISMS OF IMMUNE THERAPY RESISTANCE IN HNSCC

### Definition of immune therapy resistance

Resistance to ICIs is typically categorized into three main types based on the underlying mechanism and therapeutic timeline. (1) The first, resistance driven by target loss, arises when the TME lacks the molecular targets (e.g., PD-1/PD-L1) necessary for the antibody to engage, rendering anti-PD-1/PD-L1 monotherapy ineffective from the outset; (2) The second, primary resistance, describes a scenario where tumors, despite showing histologic evidence of immune cell infiltration and PD-L1 expression, derive no clinical benefit from initial anti-PD-1 treatment; (3) The third, acquired resistance, occurs when patients exhibit a significant initial response to ICIs, but subsequently experience disease progression during the course of therapy^[34]^. Target loss resistance refers specifically to loss of the drug target (e.g., PD-L1 downregulation), which can occur as one mechanism of acquired resistance; in contrast, acquired resistance encompasses broader mechanisms that develop after initial response to therapy, which may include target loss as one component. According to clinical manifestations, immune therapy resistance can also be categorized into four types: ① immune response not activated; ② immune response activated but suppressed by certain mechanisms; ③ pre-existing heterogeneous and resistant tumor cells before treatment; and ④ acquired resistance mutations during immune therapy^[35]^.

### Common resistance mechanisms in immune therapy for head and neck cancer

**Figure 2:**
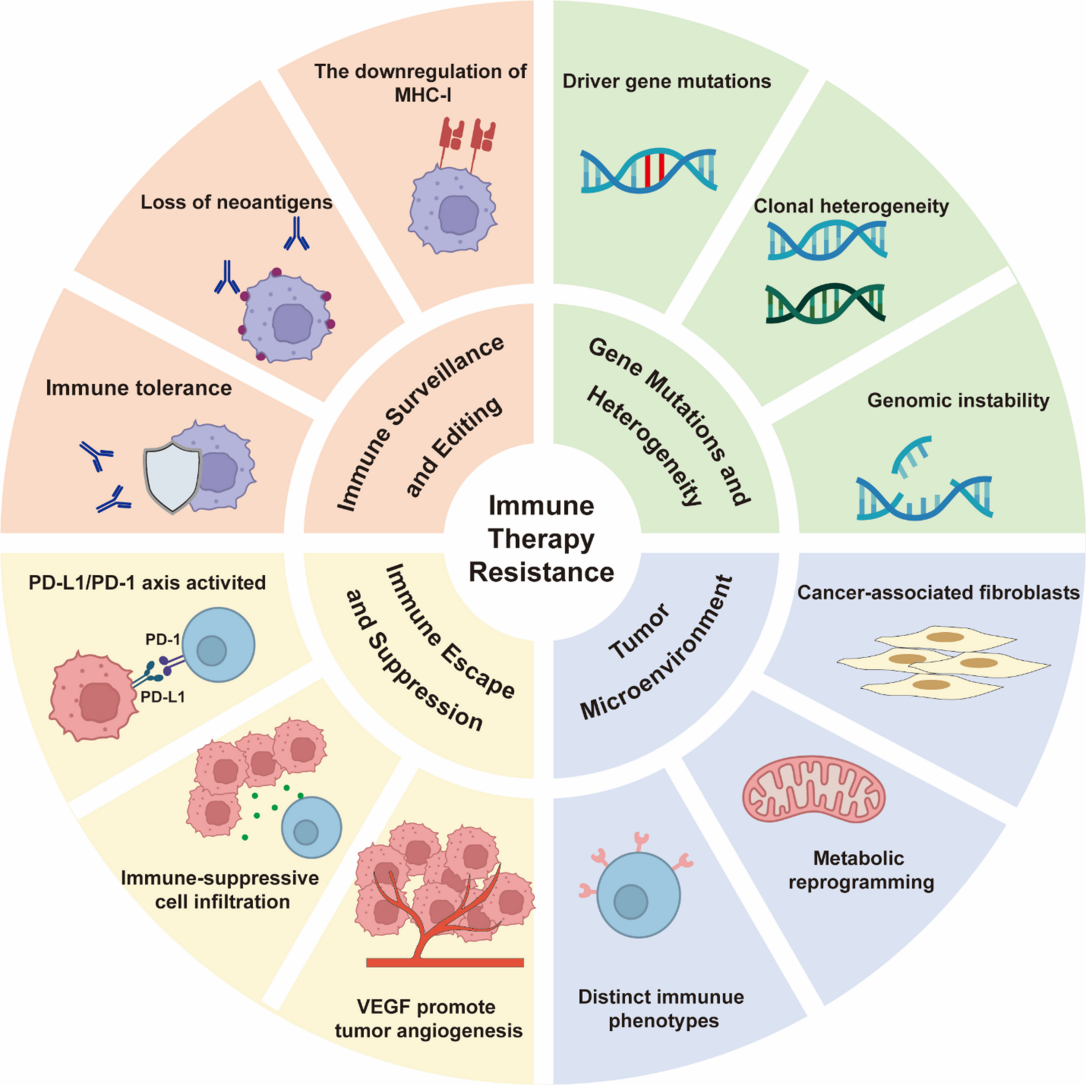
Mechanisms for immune therapy resistance in HNSCC. Created in BioRender. Yu, Y. (2025) https://BioRender.com/2oorj0t. HNSCC: Head and neck squamous cell carcinoma; MHC-1: major histocompatibility complex-1; PD-L1: programmed death-ligand 1; PD-1: programmed death-1; VEGF: vascular endothelial growth factor.

While ICIs, particularly mAbs targeting the PD-1/PD-L1 axis, have revolutionized oncology by providing durable responses in a subset of patients, their clinical benefit remains limited by a low ORR of 15%-20%^[35]^; a primary challenge is the development of resistance, which significantly curtails long-term survival benefits. The underlying mechanisms are multifaceted, involving a complex interplay between tumor-intrinsic factors, host immunity, and a suppressive TME [Figure 2].

#### Cancer immune surveillance and immune editing

The theory of cancer immune surveillance posits that the immune system maintains body homeostasis by recognizing and clearing tumor cells, but tumors can escape this process through immune editing^[36]^. Immune editing consists of three stages: elimination, equilibrium, and escape. In HNSCC, immune editing primarily promotes resistance in the following mechanisms: (1) Antigen presentation defects: Tumor cells downregulate the expression of major histocompatibility complex I (MHC-I) or antigen-processing related transporter (TAP) to reduce the presentation of tumor-associated antigens (TAAs), thereby evading immune recognition^[37]^. For example, the loss of MHC-I in HNSCC is significantly associated with resistance to PD-1 inhibitors^[38]^; (2) Loss of immunogenic antigens: Under immune pressure, tumor cells selectively lose highly immunogenic antigens (such as HPV-related E6/E7 proteins) through epigenetic silencing or gene mutations, preventing recognition by T-cells^[39]^; (3) Induction of immune tolerance: Tumor cells release immunosuppressive factors [such as interleukin (IL)-10 and transforming growth factor-beta (TGF-β)] that induce the expansion of regulatory T cells (Tregs), which suppress the function of effector T cells^[40]^.

#### Immune escape and immune suppression

The limited efficacy of immunotherapies in HNSCC can be attributed to a multifaceted network of immunosuppressive mechanisms operating within the TME. A well-characterized strategy employed by tumor cells to bypass the cytotoxic activity of T cells involves the high expression of PD-L1, which is often more pronounced in HPV-associated HNSCC. Ligation of PD-1 by PD-L1 effectively induces a state of functional exhaustion in antigen-specific T cells^[13]^. The observation that therapeutic outcomes are not consistently predicted by PD-L1 expression levels alone suggests that resistance may be driven by the engagement of other inhibitory immune checkpoints, including cytotoxic T-lymphocyte-associated protein 4 (CTLA-4), lymphocyte activation gene-3 (LAG-3), and T cell immunoglobulin and mucin domain-containing protein 3 (TIM-3)^[41]^. Beyond direct checkpoint interaction, the immunosuppressive niche is critically shaped by infiltrating myeloid cells. Tumor-associated macrophages (TAMs) suppress T-cell function through the release of soluble factors such as IL-10 and arginase-1. In parallel, myeloid-derived suppressor cells (MDSCs) induce T-cell apoptosis via the production of reactive oxygen species (ROS) and nitric oxide, creating a formidable barrier to an effective anti-tumor immune response^[42]^.

A fundamental distinction in the mechanisms of resistance to PD-1 *vs.* PD-L1 inhibitors stems from their differential binding specificities and consequent biological impacts. PD-1 inhibitors, including nivolumab and pembrolizumab, function by directly binding to the PD-1 receptor on T cells. This action provides broad-spectrum inhibition by preventing the receptor’s engagement with both of its known ligands, PD-L1 and PD-L2. The comprehensive blockade of PD-1 signaling can, however, trigger a compensatory feedback mechanism, leading to the pronounced upregulation of alternative co-inhibitory receptors such as LAG-3 and TIM-3 on exhausted T-cell populations^[41,43]^. Additionally, resistance to PD-1 inhibitors frequently correlates with T-cell-intrinsic alterations, such as JAK1/2 mutations that disrupt IFN-γ signaling and reduce antigen presentation^[35]^.

Conversely, PD-L1 inhibitors (e.g., atezolizumab, durvalumab) bind specifically to PD-L1 on tumor or stromal cells, leaving the PD-1/PD-L2 axis intact. This allows persistent PD-L2-mediated immunosuppression through alternative receptors such as repulsive guidance molecule B (RGMb), promoting adaptive resistance^[44]^. Tumor cells may also exploit PD-L1-independent pathways, including CD80 transendocytosis, which diminishes costimulatory signals for T-cell activation^[45]^. TME further differentiates resistance: PD-L1 inhibitors are more susceptible to resistance driven by MDSCs expressing PD-L2 or V-domain Ig suppressor of T cell activation (VISTA), whereas PD-1 resistance is more associated with regulatory T-cell infiltration^[46]^.

Spatial heterogeneity amplifies these differences. PD-L1 expression is highly variable within tumors, and PD-L1 inhibitors require direct target engagement on cancer cells, leading to resistance from “immune deserts” lacking T-cell infiltration or PD-L1-negative clones that evade therapy^[47]^. In contrast, PD-1 inhibitors act on circulating and tumor-infiltrating lymphocytes, making efficacy dependent on systemic T-cell functionality but vulnerable to peripheral tolerance mechanisms^[48]^. Overcoming these resistance mechanisms may thus require tailored strategies - LAG-3/TIM-3 co-inhibition for PD-1 resistance, and targeting PD-L2 or myeloid compartments for PD-L1 resistance^[49]^.

Besides, the immunosuppressive role of vascular endothelial growth factor (VEGF) extends beyond angiogenesis to include direct and indirect modulation of the immune landscape. VEGF signaling coordinates a complex immunosuppressive program by inhibiting dendritic cell (DC) maturation, reducing T-cell infiltration, and inducing inhibitory cells such as Tregs and MDSCs^[50]^. At the vascular level, VEGF-A-induced abnormal angiogenesis generates chaotic, leaky vessels with poor perfusion, leading to persistent hypoxia. This stabilizes hypoxia-inducible factor alpha (HIF-1α), which upregulates PD-L1 expression on tumor-associated endothelial cells and malignant cells, fostering an environment that excludes effector immune cells and facilitates immune evasion^[51]^. Concurrently, VEGF inhibits the activation of nuclear factor kappa-B (NF-κB) in hematopoietic progenitor cells, preventing DC differentiation. This leads to a reduced number of functional DCs, thus weakening the antigen - presenting ability and subsequent activation of T cells against tumor cells^[52]^. These mechanisms collectively establish VEGF as a non-redundant orchestrator of ICI resistance beyond its angiogenic role.

#### Gene mutations and heterogeneity in tumor cells

The high mutational burden in HNSCC should theoretically enhance immunogenicity, but gene heterogeneity and clonal evolution lead to resistance in several ways: (1) Driver gene mutations: Mutations in tumor protein 53 (TP53), notch receptor 1 (NOTCH1), and related genes inhibit T-cell infiltration by activating the wingless-int (WNT)/β-catenin pathway^[53]^. Phosphatidylinositol-4,5-bisphosphate 3-kinase catalytic subunit alpha (PIK3CA) mutations promote PD-L1 expression, leading to an immunosuppressive phenotype^[54]^; (2) Clonal heterogeneity: Multiple subclones exist within the tumor, and some subclones carrying immune escape-related mutations [e.g., Janus kinase 1/2 (JAK1/2) deletions] are selectively expanded under treatment pressure^[55]^; (3) Genomic instability: Chromosomal instability (CIN) weakens the innate immune response by inducing defects in interferon signaling pathways [e.g., inactivation of the stimulator of interferon response CGAMP interactor 1 (STING) pathway]^[56]^.

#### Impact of TME

TME is a complex ecosystem composed of tumor cells, diverse stromal cells (including immune cells, fibroblasts, endothelial cells, *etc.*), and the extracellular matrix (ECM), which collectively play a critical role in the pathogenesis, progression, metastasis, and treatment response of HNSCC. The cellular components of the TME exhibit significant heterogeneity; for instance, CD8^+^ CTLs serve as the principal anti-tumor effector cells; however, their functional capacity is frequently impaired due to exhaustion. In contrast, Tregs primarily exert immunosuppressive activities. Moreover, TAMs and MDSCs facilitate tumor immune escape through the secretion of immunosuppressive mediators. The spatial imbalance in the distribution of these cellular components and their functional phenotypes - termed the spatial heterogeneity of the TME - represents a key determinant of therapeutic resistance, particularly to interventions such as ICIs, in HNSCC.

Cancer-associated fibroblasts (CAFs) are pivotal components of TME that actively contribute to immune evasion by secreting TGF-β, a process that directly suppresses T cell infiltration into tumor sites. TGF-β signaling in CAFs promotes the creation of physical and molecular barriers, which exclude CTLs from penetrating tumor nests and accessing cancer cells, thereby limiting effective anti-tumor immune responses^[57,58]^. Specifically, CAF-derived TGF-β enhances the secretion of immunosuppressive factors and chemokines, such as C-X-C motif chemokine ligand 12 (CXCL12), that coat tumor cells and establish a milieu permissive for immune quiescence^[59]^. This secretion further recruits Tregs, amplifying the immunosuppressive microenvironment through increased TGF-β levels and fostering Treg infiltration, which collectively exhausts and excludes effector T cells^[60]^. Consequently, the TGF-β-dependent signaling in CAFs represents a key axis for modulating immune dynamics, highlighting its potential as a therapeutic target to overcome T cell exclusion and enhance immunotherapy efficacy in solid tumors.

Metabolic reprogramming, a hallmark of cancer and immune disorders, involves a shift from oxidative phosphorylation to glycolysis, leading to significant lactate accumulation in the TME^[61]^. This excessive lactate buildup creates an acidic immunosuppressive milieu that directly impairs CTL function by interfering with metabolic pathways in immune cells, promoting CTL exhaustion and dysfunction^[62]^. Specifically, lactate accumulation alters nutrient uptake and intracellular metabolic signaling in CTLs, disrupting their differentiation and activation^[63]^. Mechanistically, lactate accumulation facilitates lactylation, a post-translational modification that modifies both histone and non-histone proteins, regulating gene expression through epigenetic changes such as histone H3 lysine 18 lactylation (H3K18la). This reshapes transcriptional programs in immune cells and suppresses CTL-mediated immunity^[64]^. Lactate-derived lactylation acts as a key regulator in the immunosuppressive TME, reinforcing CTL exhaustion and limiting their persistence^[65]^. Such metabolic-epigenetic crosstalk highlights how lactate not only functions as a metabolic byproduct but also orchestrates immunosuppressive signaling that inhibits CTL efficiency, presenting a therapeutic target for overcoming immunosuppression.

Tumor heterogeneity in the TME is characterized by distinct immune phenotypes, including immune-inflamed tumors with abundant T cell infiltration and immune-desert tumors with a paucity of CD8^+^ T cells, leading to differential therapeutic outcomes. Immune-inflamed tumors are more responsive to immune checkpoint blockade (ICB) but can develop resistance through immunosuppressive niches formed by CAFs and macrophages, which spatially exclude CD8^+^ T cells and contribute to local immune evasion^[66]^. In contrast, immune-desert tumors exhibit innate resistance to ICB due to an immune-deficient microenvironment incapable of initiating effective anti-tumor responses^[67-69]^. Spatial heterogeneity, influenced by metabolic pressures and stromal interactions, promotes the emergence of drug-resistant clones and immune escape. For instance, the dynamic interplay between tumor cells and the ECM spatially restricts immune cell access, while desert regions correlate with non-expanded T cell receptor (TCR) clones and reduced MHC-I expression, exacerbating therapeutic resistance^[70-72]^. Comprehensive spatial analysis underscores the need for personalized strategies, such as enhancing immune cell infiltration in desert microenvironments or disrupting immunosuppressive niches in inflamed tumors, to overcome resistance^[73,74]^. Addressing this heterogeneity is critical for developing innovative immunomodulatory strategies.

Resistance to ICIs in HNSCC stems from multifactorial mechanisms, including tumor-intrinsic genomic heterogeneity, immunosuppressive TME dynamics, and compensatory immune checkpoint upregulation. Spatiotemporal mapping of resistance mechanisms via single-cell and spatial transcriptomics will be critical to identify context-specific therapeutic vulnerabilities for personalized interventions.

## STRATEGIES TO OVERCOME IMMUNE THERAPY RESISTANCE

**Figure 3:**
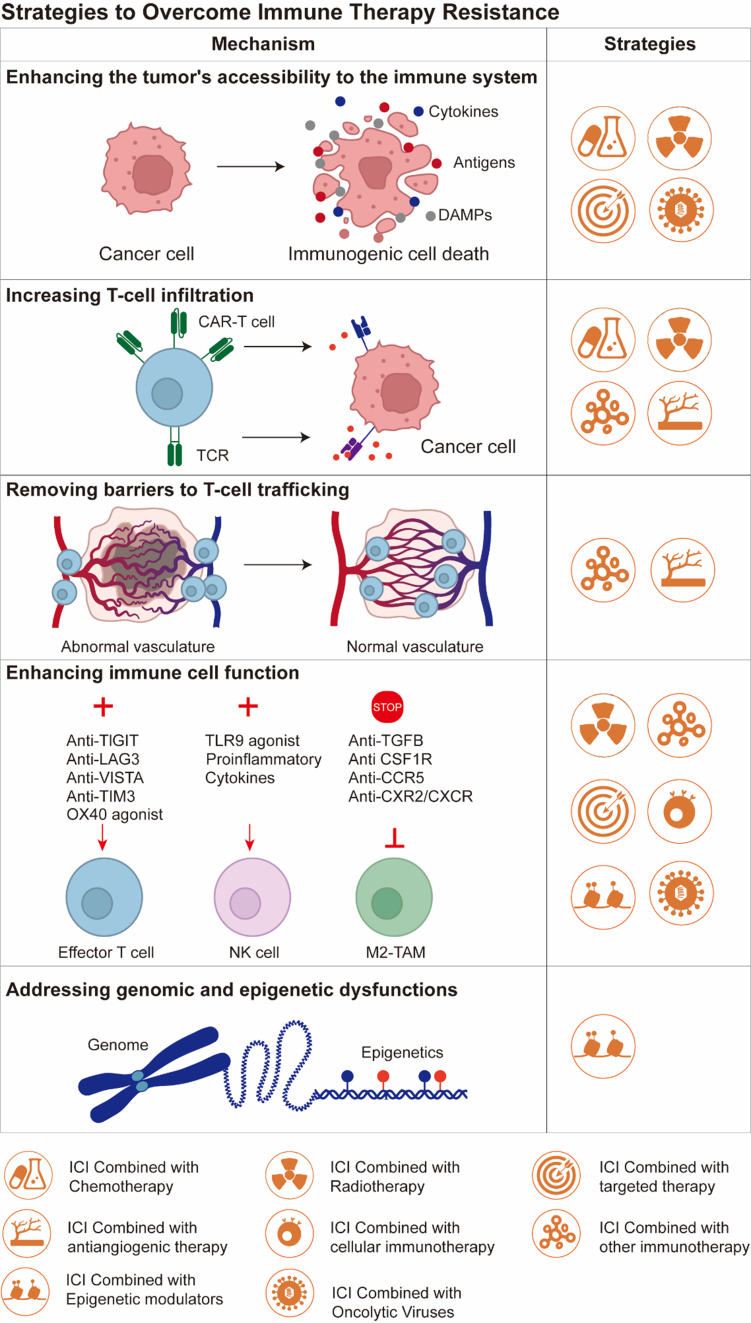
Therapeutic strategies and related mechanisms to overcome immune therapy resistance in HNSCC. Created in BioRender. Yu, Y. (2025) https://BioRender.com/2sr7zb0. HNSCC: Head and neck squamous cell carcinoma; DAMPs: damage associated molecular patterns; CAR-T cell: chimeric antigen receptor T cells; TCR: T cell receptor; TIGIT: T cell immune receptor with Ig and ITIM domains (Ig: immunoglobulin domain; ITIM: immunoreceptor tyrosine-based inhibitory motif); LAG3: lymphocyte activation gene 3; VISTA: V-domain Ig suppressor of T cell activation; TIM3: T cell immunoglobulin and mucin domain-containing protein 3; TLR9: Toll-like receptor 9; TGFB: transforming growth factor beta; CSF1R: colony-stimulating factor 1 receptor; CCR5: C-C motif chemokine receptor 5; CXR2: C-X-C motif chemokine receptor 2; CXCR: C-X-C motif chemokine receptor; NK cell: natural killer cell; M2-TAM: M2 tumor-associated macrophages; ICI: immune checkpoint inhibitor.

For patients with intrinsic and adaptive resistance to PD-1 inhibitors, more effective treatment methods need to be developed to reverse this phenomenon. Several mechanisms have been proposed to overcome immune resistance and improve patient prognosis: (I) Enhancing the tumor’s accessibility to the immune system; (II) Increasing T-cell infiltration within the tumor; (III) Removing barriers to T-cell trafficking within the tumor; (IV) Enhancing T/NK cell function; (V) Addressing genomic and epigenetic dysfunctions^[75]^. The key to overcoming immune therapy resistance lies in combining other drug targets to promote anti-tumor responses [Figure 3].

### Chemotherapy

Increasing tumor visibility to the immune system is critical for improving the efficacy of immune therapies. A major therapeutic strategy is inducing tumor cell death through cytotoxic chemotherapy^[76]^. After cell death, a large number of tumor antigens, pro-inflammatory cytokines, damage-associated molecules, calreticulin, adenosine triphosphate (ATP), *etc.*, are released into the TME, recruiting APCs and triggering subsequent T-cell activation^[77,78]^.

Chemotherapeutic agents exhibit cytotoxic effects on Tregs and MDSCs within the TME - two cell populations commonly recognized for their immunosuppressive properties^[76]^. In the treatment of HNSCC, cisplatin exerts its anti-tumor effects not only through direct cytotoxicity but also by modulating the host immune system. It enhances type I interferon and IFN-γ signaling, leading to the upregulation of PD-L1, and increases the permeability of granzyme B released by CTLs, thereby improving immune-mediated cell killing^[79,80]^. At the molecular level, chemotherapy-induced immunogenic cell death (ICD) exposes calreticulin on the cell surface and releases ATP and high mobility group box 1 (HMGB1), which enhance DC maturation and antigen presentation^[81]^.

Advanced delivery platforms (e.g., ROS-responsive nanosystems) further optimize synergy by spatial-temporal coordination of drug release and immune activation^[82]^. This multimodal approach addresses the complexity of multidrug resistance (MRD) by concurrently targeting tumor cell-intrinsic resistance mechanisms (e.g., ferroptosis evasion^[83]^) and extrinsic immunosuppressive factors. The integration of chemotherapy’s direct cytotoxicity with immunotherapy’s immune memory effects creates a positive feedback loop that sustains tumor suppression.

### Radiotherapy

Radiotherapy kills cancer cells mainly through two mechanisms: high-energy radiation directly affects intracellular DNA or induces ROS. Radiation causes DNA single- or double-strand breaks, leading to apoptosis if the tumor cells cannot properly repair the damaged genetic material. Radiotherapy can upregulate immune responses locally or systemically, indirectly inducing anti-tumor immune responses, which can affect immune therapy. Current research suggests that the immune response induced by radiotherapy involves several key mechanisms: (I) Ionizing radiation triggers ICD, stimulating the recruitment and differentiation of tumor-specific T lymphocytes^[84]^; (II) Radiotherapy activates the cyclic GMP-AMP synthase (cGAS; GMP: guanosine monophosphate, AMP: adenosine monophosphate)-STING signaling pathway, initiating type I interferon responses^[85,86]^; (III) Radiotherapy enhances the expression of antigen-presenting molecules on tumor cells^[87,88]^; (IV) Radiotherapy promotes the release of inflammatory factors, remodeling the tumor immune microenvironment^[89,90]^; (V) Radiotherapy upregulates the expression of immune checkpoints and death receptors on tumor cells^[84]^. There are some clinical trials involving the combination of radiotherapy and immunotherapy for HNSCC, mostly prospective phase I/II studies and retrospective analyses [Table 3]. The integration of ICIs with radiotherapy in HNSCC has yielded mixed outcomes across clinical trials. In the NCT02609503 trial, which enrolled 29 platinum-ineligible stage III/IV patients, concurrent pembrolizumab and radiotherapy led to a 2-year PFS of 71% and OS of 75%. However, grade 3-4 lymphopenia occurred in 59% of participants, alongside typical radiation-related toxicities^[91]^. Conversely, the phase III KEYNOTE-412 (NCT03040999) study demonstrated that adding pembrolizumab to chemoradiotherapy failed to significantly improve event-free survival (EFS) compared to placebo in an unselected locally advanced HNSCC population. High-grade adverse events (AEs) were frequent in both groups (92% with pembrolizumab *vs.* 88% with placebo), with no new safety signals identified^[15]^. Similarly, the NCT02952586 trial reported no significant PFS or OS benefit from adding avelumab to cisplatin-based radiotherapy^[92]^. The timing, radiotherapy dosage/fractionation regimen, and potential beneficiary populations of radiotherapy combined with immunotherapy need further validation through more clinical trials.

**Table 3:** Clinical trials of combination therapy with chemoradiotherapy in HNSCC

**Trial name**	**Phase**	**Comparison**	**Enrolled patients**	**Outcome measures**
NCT02609503^[91]^	II	Radiotherapy + pembrolizumab	29	PFS ≥ 16 months
NCT03040999^[15]^	III	Chemoradiotherapy ± pembrolizumab	804	EFS
NCT02952586^[92]^	III	Chemoradiotherapy ± avelumab	697	PFS

HNSCC: Head and neck squamous cell carcinoma; PFS: progression-free survival; EFS: event-free survival.

### Combination with other immune therapies

#### CTLA-4 inhibitors

Simultaneously blocking inhibitory checkpoints or activating stimulatory pathways can maximally enhance effector T-cell function [Table 4]. Although the combination of anti-PD-1/PD-L1 and anti-CTLA-4 agents has demonstrated superior efficacy over monotherapy in multiple tumor types, its clinical benefits in specific malignancies such as HNSCC require further precise delineation. The mechanistic basis for this synergy stems from the competitive binding of CTLA-4 to cluster of differentiation 80/cluster of differentiation 86 (CD80/CD86) ligands on APCs, which attenuates the costimulatory signals delivered through cluster of differentiation 28 (CD28) and consequently suppresses the initial activation of T cells. In contrast, anti-CTLA-4 antibodies not only block ligand engagement by binding CTLA-4 with high affinity but may also deplete or functionally inhibit Tregs within the TME, thereby alleviating immunosuppression and potentiating anti-tumor immunity. Nonetheless, clinical outcomes have been variable; for instance, the phase III EAGLE trial (NCT02369874) in R/M HNSCC revealed no statistically significant improvement in OS with durvalumab (anti-PD-L1) plus tremelimumab (anti-CTLA-4) compared to durvalumab monotherapy^[93]^. Beyond the PD-1/PD-L1 and CTLA-4 pathways, other co-inhibitory receptors - including TIM-3, LAG-3, and T cell immune receptor with Ig and ITIM domains (TIGIT; Ig: immunoglobulin domain, ITIM: immunoreceptor tyrosine-based inhibitory motif), which are expressed on tumor-infiltrating lymphocytes - have emerged as promising next-generation targets for reversing T-cell exhaustion and reinvigorating anti-tumor immune responses.

**Table 4:** Clinical studies of other immunotherapeutic approaches in HNSCC

**Agent**	**Description**	**Phase**	**Therapeutic comparison**	**Target drug-resistant types**	**Sponsor**
Tremelimumab^[93]^	mAb to human CTLA-4	III	Durvalumab	Synergistically activate immune initiation; overcome chemotherapy resistance; transform the “cold tumor” microenvironment	AstraZeneca
Relatlimab^[94]^	mAb to human LAG-3	II	Nivolumab	Compensatory LAG-3 upregulation; T cell exhaustion	Bristol-Myers Squibb
TSR-033^[95]^	mAb to human LAG-3	I	Dostarlimab (anti-PD-1) and Cobolimab (anti-TIM-3) plus Bevacizumab (anti-VEGFA) and FOLFIRI (folinic acid/leucovorin, 5-FU and irinotecan)	Compensatory LAG-3 upregulation; T cell exhaustion	Tesaro
Fianlimab^[96]^	mAb to human LAG-3	I	Cemipilimab (anti-PD-1)	Compensatory LAG-3 upregulation; T cell exhaustion	Regeneron/Sanofi
BI754111^[97]^	mAb to human LAG-3	II	Ezabenlimab (anti-PD-1)	Compensatory LAG-3 upregulation; T cell exhaustion	Boehringer Ingelheim
FS118^[98]^	Bispecific anti-LAG-3-anti-PD-L1	I/II	N/A	CD8^+^ TIL cells exhausted	F-Star
XmAb22841^[99]^	Bispecific LAG-3/CTLA-4 antibody	I	N/A	T cell exhaustion; Treg-mediated TME inhibition	Xencor
Tebotelimab^[100]^	DART anti-LAG-3-anti-PD-L1	II	Enoblituzumab (anti-B7-H3)	CD8^+^ TIL cells exhausted	MacroGenics
Eftilagimod alpha^[101]^	LAG-3-Ig agonist	II	Pembrolizumab (anti-PD-1); avelumab (anti-PD-L1)	Primary drug resistance in PD-L1 low expression/negative tumors; acquired resistance following PD-1/PD-L1 inhibitor therapy	Immutep
Sabatolimab^[102]^	mAb to human TIM-3	I/Ib	Spartalizumab (anti-PD-1)	Inhibition of T cells and NK cells	Novartis
RO-7121661^[103]^	Bispecific anti-TIM-3-anti-PD-L1	I	N/A	T cell exhaustion; TIM-3 is compensatorily upregulated, establishing bypass immunosuppression	Roche
AZD-7789^[104]^	Bispecific anti-TIM-3-anti-PD-L1	I/IIa	N/A	T cell exhaustion; TIM-3 is compensatorily upregulated, establishing bypass immunosuppression	AstraZeneca
Tiragolumab^[105]^	mAb to human TIGIT	II	Atezolizumab (anti-PD-L1)	Compensatory upregulation of TIGIT; remodel the tumor immune microenvironment	Roche
OMP-313M32^[106]^	mAb to human TIGIT	I	Nivolumab (anti-PD-1)	Compensatory upregulation of TIGIT; remodel the tumor immune microenvironment	Mereo BioPharma 5
Enoblituzumab^[100]^	B7-H3 inhibitor	I/II	Pembrolizumab (anti-PD-1)	Immunosuppressive function of B7-H3	MacroGenics
SD-101^[107]^	Synthetic CpG-ODN agonist of TLR9	Ib/II	Pembrolizumab (anti-PD-1)	T-cell exhaustion and compensatory immunosuppression	Dynavax Technologies
GSK3359609^[108]^	ICOS agonist antibody	III	Pembrolizumab (anti-PD-1)	PD-L1 low expression/negative tumor; T cell exhaustion or immunosuppressive microenvironment	GlaxoSmithKline

HNSCC: Head and neck squamous cell carcinoma; mAb: monoclonal antibody; CTLA-4: cytotoxic T-lymphocyte-associated protein 4; LAG-3: lymphocyte activation gene-3; PD-1: programmed death-1; TIM-3: T cell immunoglobulin and mucin domain-containing protein 3; VEGFA: vascular endothelial growth factor A; 5-FU: 5-fluorouracil; CD8^+^: cluster of differentiation 8 positive; TIL: tumor immune infiltration; TME: tumor microenvironment; PD-L1: programmed death-ligand 1; NK: natural killer; TIGIT: T cell immune receptor with Ig and ITIM domains (Ig: immunoglobulin domain; ITIM: immunoreceptor tyrosine-based inhibitory motif); B7-H3: B7 homolog 3 protein; TLR9: Toll-like receptor 9; ICOS: inducible T-cell costimulatory.

#### LAG-3 inhibitors

LAG-3, an immunoinhibitory receptor structurally homologous to CD4, is upregulated on T cells following activation and maintains persistent expression under conditions of chronic antigen exposure, such as cancer and persistent viral infections^[109-111]^. Its expression extends beyond T cells to encompass various immune cells, including NK cells, B cells, and DCs. On conventional T cells, LAG-3 functions as a critical negative regulator, suppressing T-cell activation and cytokine production, thereby contributing to a state of T-cell exhaustion^[112]^. Additionally, LAG-3 is expressed on forkhead box P3 positive (FOXP3^+^) Treg cells, leading to an immunosuppressive state^[113]^. Although LAG-3 blockade alone did not show benefits in preclinical studies, when combined with PD-1 inhibitors, it significantly enhanced tumor control in tumor models. The pivotal RELATIVITY-047 trial (NCT03470922), a global, randomized, double-blind phase II/III study, evaluated the combination therapy Opdualag (anti-LAG-3 plus anti-PD-1) *vs.* nivolumab monotherapy in patients with unresectable or metastatic melanoma. The results demonstrated a superior median PFS of 10.1 months for the Opdualag group compared to 4.6 months for the nivolumab group. The safety profile was manageable, with common immune-related AEs (irAEs) including thyroiditis and rash^[114]^. Based on these findings, the U.S. FDA granted approval for this combination in 2022. Consequently, multiple phase I and II clinical trials are ongoing to further explore the efficacy of LAG-3 inhibitors, both as monotherapy and in combination regimens, in other malignancies, including HNSCC^[43]^.

#### TIM-3 inhibitors

TIM-3, a member of the T cell immunoglobulin mucin (TIM) family of immunoregulatory proteins, functions as a multifaceted inhibitory receptor within the TME. Initially characterized as a marker for IFN-γ-producing CD4^+^ (Th1) and CD8^+^ T cells, subsequent research has revealed that its expression extends to a diverse array of immune cells^[115]^. These include Tregs, MDSCs, NK cells, and mast cells, indicating that TIM-3-targeted therapeutic strategies may exert immunomodulatory effects through multiple cellular pathways^[116-118]^. Preclinical investigations have demonstrated that concurrent blockade of the TIM-3 and PD-1 pathways can induce significant tumor regression, a finding that has accelerated the clinical translation of anti-TIM-3 agents^[119,120]^. Among the most advanced candidates are sabatolimab (MBG453) and cobolimab (TSR-022). Sabatolimab has received Fast Track designation from the FDA for use in combination with hypomethylating agents (HMAs) for the treatment of myelodysplastic syndromes (MDS)^[121]^. Meanwhile, cobolimab is under evaluation in phase II/III clinical trials for various solid tumors, including lung cancer, hepatocellular carcinoma, and melanoma, exploring its efficacy in combination with PD-1 inhibitors and/or chemotherapeutic drugs. Encouragingly, a phase I trial investigating cobolimab in combination with a PD-1 inhibitor for lung cancer reported an ORR of 42.9%, alongside a favorable tolerability profile without serious treatment-emergent AEs^[122]^.

#### TIGIT inhibitors

TIGIT, a newly identified co-inhibitory receptor, was discovered in 2009^[123]^. Its expression pattern varies across immune cell populations: it is transiently induced on T cells following TCR stimulation, while being constitutively expressed on NK cells and multiple T-cell subsets, such as Tregs, type 1 regulatory T (Tr1) cells, follicular helper T (Tfh) cells, and functionally impaired CD8^+^ T cells^[124]^. Emerging evidence has further demonstrated TIGIT expression on regulatory B cells and innate immune cell populations^[125]^, suggesting that this receptor may exert regulatory effects in immune responses that extend beyond its well-documented roles. Upon engagement with its cognate ligands, TIGIT exerts suppressive effects on T-cell activation and impairs NK cell functionality^[126]^. Moreover, within the TME, TIGIT mediates tumor immune escape through dual mechanisms: direct inhibition of effector CD8^+^ T cells and augmentation of the immunosuppressive properties of Tregs^[127]^. A current phase II trial (CITYSCAPE-02, NCT03563716) is comparing the efficacy of PD-L1 inhibitors alone *vs.* a combination of TIGIT inhibitors with PD-L1 inhibitors in NSCLC patients. Results showed that the combination therapy group had a significantly higher ORR compared to the monotherapy group, with both PFS and OS improving^[128]^. Besides NSCLC, other cancers are also being tested for the potential of TIGIT blockade as a therapeutic target. In the SKYCRAPER-09 trial (NCT03563716), the efficacy of tiragolumab combined with atezolizumab in R/M HNSCC was compared with a placebo^[129]^. Additionally, multiple phase II/III clinical trials are ongoing in lung cancer, liver cancer, kidney cancer, gastrointestinal tumors, and HNSCC with anti-TIGIT/anti-PD-1/anti-PD-L1 combined with chemotherapy, and we look forward to the results of these trials in the future^[49]^.

#### B7 homolog 3 protein inhibitors

B7 homolog 3 protein (B7-H3) is a dual-function immune checkpoint molecule with both costimulatory and co-inhibitory properties, whose expression spans cancer cells and bone marrow-derived immune cells - including T lymphocytes, B lymphocytes, monocytes/macrophages, and DCs^[130]^. Notably, elevated B7-H3 expression levels in tumor cells and immune-infiltrating cells within the TME have been linked to the development of tumor immune evasion^[131]^. Emerging evidence suggests that B7-H3 promotes tumor proliferation, metastasis, and therapeutic resistance. In oral squamous cell carcinoma, B7-H3 overexpression is linked to increased tumor aggressiveness and higher mortality rates, with blockade suppressing tumor formation^[132]^. These findings highlight B7-H3 as a promising therapeutic target. A phase I trial evaluated the B7-H3 inhibitor enoblituzumab combined with the anti-PD-1 inhibitor retifanlimab across multiple tumor types, showing an ORR of 33.3% in 18 HNSCC patients, including five partial responses (PRs) and one complete response (CR)^[133]^. These promising phase I results have led to subsequent phase II/III trials to assess the safety and anti-tumor activity of combination therapy.

#### Inducible T cell costimulatory agonists

The Inducible T cell costimulator (ICOS) pathway, an activation-induced T-cell costimulatory receptor, plays a critical role in modulating T-cell proliferation, survival, and differentiation^[134]^. ICOS ligand (ICOSL) is expressed by APCs and various tumor cells within the microenvironment. ICOS ligation triggers downstream signaling that exerts pleiotropic effects across T-cell subsets, potentially enhancing CD8^+^ T-cell cytotoxicity while simultaneously amplifying the immunosuppressive function of Tregs^[135]^. Although preclinical studies supported a synergistic effect between ICOS agonism and PD-1 blockade, clinical development faced challenges, as evidenced by the termination of the phase II/III INDUCE-3 (NCT04128696) and INDUCE-4 (NCT04428333) trials of the ICOS agonist feladilimab in R/M HNSCC due to insufficient efficacy^[108]^. Subsequent research has shifted toward novel bispecific antibodies, such as izuralimab (XmAb23104), which targets both PD-1 and ICOS to concurrently block inhibitory signaling and provide agonist costimulation.

#### TLR agonists

Toll-like receptor 9 (TLR9) agonists have been widely studied in both monotherapy and combination therapy for cancer treatment. Combination therapy with TLR9 agonists and immunotherapy has shown a synergistic effect, enhancing anti-tumor immune responses. TLR9 TLR9 agonists are capable of activating B lymphocytes and plasmacytoid DCs (pDCs), which in turn enhances the secretion of chemokines and cytokines. This immunomodulatory effect optimizes the tumor immune microenvironment and facilitates T cell-driven anti-tumor immune responses^[136]^. A phase Ib clinical trial (ClinicalTrials.gov: NCT02680184) investigated the therapeutic efficacy and safety profile of intratumoral administration of the Toll-like receptor (TLR) agonist Vidutolimod in combination with a PD-1 inhibitor. In this study involving 44 patients, 11 individuals achieved either a PR or CR, with a median PFS of 2.8 months. These results highlight the potential of TLR9 agonists to reverse PD-1 inhibitor resistance in patients with advanced melanoma^[137]^. Another clinical study (NCT02521870) evaluated the safety and efficacy of SD-101 - a synthetic CpG oligodeoxynucleotide (CpG-ODN) acting as a TLR9 agonist - in combination with pembrolizumab for the treatment of R/M HNSCC. Among the 28 enrolled patients, the ORR was recorded at 22%^[138]^.

### Research and development of emerging drugs or approaches

#### Epidermal growth factor receptor inhibitors

Epidermal growth factor receptor (EGFR) is an established therapeutic target for HNSCC. There is a theoretical rationale for combining EGFR inhibitors with ICIs. Preclinical studies have demonstrated that EGFR signaling can influence the TME by modulating cytokines such as IL-6, TGF-β, and progranulin, thereby suppressing immune-mediated anti-cancer effects. EGFR activation induces the expression of PD-L1 and other immunosuppressive factors^[139]^. Cetuximab is a mAb used for HNSCC treatment that inhibits EGFR signaling. The NCT01218048 study revealed that after cetuximab treatment, PD-1 expression on CD8^+^ TILs increased significantly and was closely associated with treatment response, providing a theoretical basis for combination therapy. A phase II trial evaluated pembrolizumab plus cetuximab in platinum-refractory recurrent HNSCC (33 patients). The ORR was 45%, with a median duration of response (DOR) of 14.9 months and no severe treatment-related toxicity^[140]^. Multiple phase I-II trials of PD-1/PD-L1 plus EGFR inhibitors in R/M HNSCC are complete [Table 5]; an ongoing phase III trial assesses cetuximab/radiotherapy with avelumab in locally advanced HNSCC^[146]^.

**Table 5:** Clinical studies combining immunotherapy with other targeted drugs in HNSCC

**Trial name**	**Phase**	**Comparison**	**Overcome drug-resistant types**	**Enrolled patients**	**Outcome measures**
NCT03082534^[140]^	II	Cetuximab (EGFR inhibitor) + pembrolizumab (anti-PD-1)	Cold TME; immune escape triggered by EGFR-targeted therapy	33	OS
NCT02938273^[133]^	I	Cetuximab (EGFR inhibitor) + radiotherapy + avelumab (anti-PD-L1)	Primary microenvironment-mediated resistance; compensatory immunosuppression	10	Efficacy and safety
NCT03691714^[141]^	II	Cetuximab (EGFR inhibitor) + durvalumab (anti-PD-L1)	EGFR-driven adaptive immune escape; antigen presentation-deficient resistance	Ongoing	OS
NCT03370276^[142]^	II	Cetuximab (EGFR inhibitor) + nivolumab (anti-PD-1)	Cold TME; immune escape	95	OS
NCT02501096^[143]^	Ib/II	Lenvatinib (VEGFR inhibitor) + pembrolizumab (anti-PD-1)	Vascular barrier-mediated primary resistance; clonal evolution-mediated acquired resistance	22	ORR PFS DOR
NCT04199104^[144]^	III	Pembrolizumab (anti-PD-1) ± lenvatinib (VEGFR inhibitor)	Vascular barrier-mediated primary resistance; clonal evolution-mediated acquired resistance	511	ORR PFS OS
NCT02178722^[145]^	I/II	Pembrolizumab (anti-PD-1) + epacadostat (IDO1 inhibitor)	Tryptophan metabolism-driven adaptive resistance; DC dysfunction resistance	38	ORR DCR

HNSCC: Head and neck squamous cell carcinoma; EGFR: epidermal growth factor receptor; PD-1: programmed death-1; TME: tumor microenvironment; OS: overall survival; PD-L1: programmed death-ligand 1; VEGFR: vascular endothelial growth factor receptor; ORR: overall response rate; PFS: progression-free survival; DOR: duration of response; IDO1: indoleamine-2,3-dioxygenase 1; DC: dendritic cell; DCR: disease control rate.

A meta-analysis of seven trials showed cetuximab + anti-PD-1 significantly improved ORR and 1-year OS in HPV-negative R/M HNSCC *vs.* monotherapy, but no benefit in HPV-positive disease. However, subgroup analysis indicated no significant effect in HPV-positive diseases^[147]^.

#### VEGFR inhibitors

Lenvatinib acts as a broad-spectrum receptor tyrosine kinase (RTK) inhibitor, which exerts robust inhibitory effects on major mediators of tumor angiogenesis and progression. These key regulatory targets include vascular endothelial growth factor receptors 1-3 (VEGFR1-3), fibroblast growth factor receptors 1-4 (FGFR1-4), platelet-derived growth factor receptor α (PDGFRα), as well as the KIT proto-oncogene, receptor tyrosine kinase (KIT) and ret proto-oncogene (RET) kinases. Clinically, this agent has secured approval from FDA for the treatment of several malignancies, specifically radioactive iodine-refractory differentiated thyroid carcinoma, advanced renal cell carcinoma, and unresectable hepatocellular carcinoma. The KEYNOTE-146 phase Ib/II trial (NCT02501096) assessed the efficacy and safety of pembrolizumab combined with lenvatinib in HNSCC, showing improved ORR and a median PFS of 7.6 months [95% confidence interval (CI) 4.2-12.6]. Treatment-related AEs primarily included fatigue, appetite loss, hypertension, diarrhea, and nausea, without severe toxic effects^[148]^. Based on these findings, a phase III trial (LEAP-010, NCT04199104) was initiated to evaluate pembrolizumab with or without lenvatinib in R/M HNSCC^[149]^. Results showed that in PD-L1 CPS ≥ 1 R/M HNSCC patients, the combination therapy significantly improved PFS and ORR, but OS was not prolonged. The safety profile was consistent with previous reports, although 156 (61.4%) patients in the combination therapy group experienced treatment-related AEs, compared to 45 (17.8%) in the placebo group.

In addition, multiple studies have investigated the efficacy of combining VEGFR inhibitors with ICIs in the treatment of metastatic or recurrent HNSCC. For instance, a phase 1/2 trial evaluating ramucirumab (a mAb targeting VEGFR2) in combination with pembrolizumab for recurrent or metastatic HNSCC demonstrated encouraging results. In this trial, ORR reached 55%, highlighting the superiority of the combination therapy over single-agent immunotherapy in improving response rates^[150]^. Additionally, a phase II trial of pembrolizumab combined with cabozantinib (a multi-targeted RTK inhibitor including VEGFR) achieved promising outcomes, meeting the primary endpoint of improved ORR^[151]^. Cabozantinib modulates immune cell function within the TME by targeting VEGFR and other pathways, thereby synergizing with pembrolizumab to enhance tumor control.

#### Indoleamine-2,3-dioxygenase 1 inhibitors

Indoleamine-2,3-dioxygenase 1 (IDO1), a key immunomodulatory enzyme, catalyzes the initial and rate-limiting step of tryptophan catabolism along the kynurenine pathway, thereby suppressing CTL activity and contributing to an immunosuppressive TME^[152]^. In HNSCC, elevated expression of IDO1 is frequently observed within malignant cells, as well as in TAMs and DCs, and this overexpression is correlated with unfavorable patient prognosis^[153]^. IDO1 plays a role in T cell exhaustion and inhibition while promoting Treg cell activation and MDSC infiltration^[154,155]^. The selective IDO1 inhibitor epacadostat was developed to counteract this mechanism. Studies have also suggested that IDO1 activity is closely related to ICI resistance. A phase I/II clinical trial evaluating epacadostat combined with pembrolizumab in HNSCC showed an ORR of 34%^[156]^. However, the CheckMate 9NA/ECHO-310 phase III study (NCT03342352), which assessed the same treatment combination in melanoma, yielded poor results, leading to early study termination^[157]^.

#### Antibody–drug conjugates

Antibody–drug conjugates (ADCs) are hybrid therapeutic agents composed of mAbs and small-molecule cytotoxic payloads, where the two components are covalently linked via a chemical linker. The mAb moiety of ADCs is specifically designed to bind to tumor-specific antigens (TSAs) or TAAs, enabling targeted delivery of the cytotoxic agent to cancerous cells. This unique targeted mechanism endows ADCs with two key advantages: enhanced safety profiles and an expanded therapeutic window compared to conventional chemotherapeutics. Furthermore, the antibody moiety of ADCs interacts with immune effector cells in the TME, triggering antibody-dependent cellular cytotoxicity (ADCC) to enhance anti-tumor effects^[158]^. A NCT04223856 trial evaluated Enfortumab Vedotin (ADC) plus Pembrolizumab as first-line therapy for advanced urothelial carcinoma. Results showed that the combination group had significantly longer PFS (12.5 *vs.* 6.3 months) and OS (31.5 *vs.* 16.1 months) than the standard chemotherapy group^[159]^. The combination of ADCs and ICIs may offer a new potential treatment approach for R/M HNSCC. Based on data from other cancer types, further research into this combination therapy is warranted.

#### Oncolytic viruses

Oncolytic viruses represent a distinctive category of viruses characterized by their ability to selectively proliferate within cancer cells, trigger apoptotic cell death in these malignant targets, while leaving normal, non-tumorigenic tissues intact^[160]^. RP1, a herpes simplex virus type 1 (HSV-1)-based oncolytic immunotherapeutic agent, is engineered to express two key bioactive molecules: human granulocyte-macrophage colony-stimulating factor (GM-CSF) and the chimeric fusion protein Gibbon Ape Leukemia Virus envelope glycoprotein (GALV-GP-R). This dual-expression design strengthens the eradication of tumor cells by potentiating the activation of systemic anti-tumor immune responses. The phase I/II IGNYTE study (NCT03767348) assessed the therapeutic efficacy of RP1 in combination with nivolumab among melanoma patients who had previously experienced treatment failure with PD-1 inhibitors. Study outcomes indicated that at the 12-month follow-up, the ORR reached 33.6%. Further stratification showed an objective response rate of 31.4% and a CR rate of 12.2%. Notably, the mean DOR surpassed 35 months, and the combined regimen exhibited favorable tolerability in the study cohort^[161]^. Beyond RP1, other oncolytic viruses - including vaccinia virus, vesicular stomatitis virus, and measles virus - are currently under clinical investigation for the treatment of head and neck cancer^[162,163]^.

#### Nanomedicine approaches for cancer treatment

The insufficient accumulation of ICIs within HNSCC tumors constitutes a critical barrier to their therapeutic effectiveness. This limitation is mainly attributed to aberrant tumor vascularization, elevated interstitial fluid pressure, and a dense ECM^[164]^. Nano-drug delivery systems (NDDSs) have emerged as a promising strategy for the targeted delivery of diverse ICIs, with the majority of current research focusing on PD-1/PD-L1 inhibitors. These nanosystems enable the site-specific delivery of various ICI types - including antibodies, nucleic acids, and small-molecule inhibitors - to tumor tissues, thereby boosting anti-tumor potency. Furthermore, NDDSs can be engineered for dual ICI co-delivery, such as the simultaneous transport of PD-1/PD-L1 and CTLA-4 inhibitors^[165]^. Collectively, NDDSs have not only enhanced the therapeutic efficacy of ICIs but also mitigated the incidence of irAEs. Nanomedicine approaches address the pharmacokinetic limitations that have hampered traditional combination therapies. For example, glutathione (GSH)-sensitive polyethylene glycol-poly-L-lysine (PEG-PLL) micelles are surface-modified with angiopep-2 peptide [to penetrate the blood-brain barrier (BBB)] and co-load anti-PD-L1 and paclitaxel (PTX) to achieve chemo-immunotherapy for glioma^[166]^; Hollow mesoporous organosilicon nanoparticle-coated Fe-MOF loaded with doxorubicin (DOX), which can locally release DOX after surgery to induce ICD, combined with anti-PD-1 to inhibit postoperative recurrence and brain metastasis of 4T1 breast cancer^[167]^. Man@pSiNPs-erastin (mannose-modified porous silicon nanoparticles loaded with erastin), targeting TAM to induce ferroptosis, combined with anti-PD-1 to inhibit liver cancer^[168]^; Cu_2_WS_4_-PEG nanozyme induces ferroptosis through the Kelch-like ECH associated protein 1 (KEAP1)/nuclear factor erythroid 2–related factor 2 (NRF2)/heme oxygenase 1 (HMOX1)/glutathione peroxidase 4 (GPX4) pathway, and enhances immunoradiotherapy in combination with radiotherapy and anti-PD-L1^[169]^.

In conclusion, NDDSs have unique advantages and potential in the treatment of R/M HNSCC, and various development strategies can help them maximize therapeutic effects and reduce side effects in treatment.

### Emerging therapeutic paradigms: beyond conventional combinatorial approaches

#### Neoadjuvant ICB

Neoadjuvant ICIs leverage intact tumor antigenicity to prime systemic anti-tumor immunity prior to surgery. This approach induces *in situ* formation of tertiary lymphoid structures (TLS) and clonal T-cell expansion, enhancing micrometastasis eradication. TLS are lymph node-like structures formed in chronic inflammatory or TMEs. While not endogenously formed at birth, these structures exhibit functional similarities to lymph nodes: they recruit immune cell populations - including T lymphocytes, B lymphocytes, and DCs - and facilitate localized immune responses. Within the TLS microenvironment, DCs capture TAAs and present them to naive T cells. Subsequently, the activated T cells undergo clonal proliferation under the stimulation of cytokines (e.g., IL-2), yielding a large pool of effector T cells that share identical antigenic specificity. This process enhances the recognition and cytotoxic capacity against tumor cells. In tumors, the presence of TLS is often correlated with improved immune infiltration and better prognosis, as they provide an organized immunological niche where immune cells can be activated and proliferate. For resectable HNSCC patients undergoing neoadjuvant pembrolizumab therapy, the surgical procedure was successfully accomplished in roughly 88% of participants across all study arms. The median duration of follow-up stood at 38.3 months. With respect to 36-month EFS outcomes, distinct differences were observed between the pembrolizumab and control groups: in the population with a CPS of 10 or higher, the EFS rates were 59.8% and 45.9%, respectively; whereas in the CPS ≥ 1 subgroup, the rates were 58.2% *vs.* 44.9%; and in the total population, 57.6% *vs.* 46.4%^[170]^. Mechanistically, ICIs expanded tumor-reactive T-cell clones post-surgery, reduced MDSCs, and induced systemic CD8^+^ memory differentiation driving abscopal effects^[171]^. Phase III trials (e.g., KEYNOTE-689, NCT03765918) now validated pathologic response as a surrogate endpoint for OS^[172,173]^.

#### Microbiome modulation: the gut-immune axis as a therapeutic target

Based on provided literature, the gut microbiome significantly modulates ICI efficacy in cancers, including HNSCC, through intricate mechanisms involving immune crosstalk: (1) Molecular Mimicry: Bacterial peptides can structurally resemble tumor antigens. For instance, peptides derived from Bacteroides fragilis exhibit cross-reactivity with TP53 antigens^[174]^. This mimicry potentially triggers or modifies T-cell responses targeting tumors; (2) Metabolite Production: Microbial metabolites profoundly influence immune cell differentiation and function. Short-chain fatty acids (SCFAs), produced by commensal bacteria during fiber fermentation, promote the differentiation and function of Tregs^[175]^. Conversely, the microbial metabolite inosine enhances anti-tumor Th1 responses, potentiating ICI activity^[176]^; (3) DC Priming: Specific commensal bacteria directly prime DCs, essential for initiating adaptive immunity. Akkermansia muciniphila, a species associated with positive ICI responses, enhances DC function leading to the upregulation of IL-12. This, in turn, promotes the recruitment and activation of cytotoxic CD8^+^ T cells within the TME^[177]^.

Clinically, modulation strategies such as Fecal Microbiota Transplantation (FMT) exploit these mechanisms to overcome resistance. Transplanting microbiota from ICI-responsive donors to refractory patients enriches beneficial taxa (e.g., Ruminococcaceae) and has demonstrated the capacity to double objective response rates compared to control groups (32% *vs.* 15%) in refractory cancers^[178]^. Importantly, the microbiome’s impact extends beyond the gut. Within the HNSCC context, a high abundance of Fusobacterium in the salivary microbiome has been negatively correlated with resistance to PD-1 inhibitors, suggesting that modulating both gut and local oral/respiratory microbiota represents a viable therapeutic avenue to enhance ICI efficacy and circumvent resistance^[179]^. Collectively, targeting the microbiome-immune axis is emerging as a critical strategy to optimize immunotherapy outcomes.

#### Multimodal therapeutic innovations

CAR-NK cell therapy: precision off-the-shelf immunity Chimeric antigen receptor-modified NK (CAR-NK) cells are a pivotal breakthrough for overcoming solid tumor resistance. Via natural cytotoxicity receptors, they exert MHC-unrestricted cytotoxicity, and their PD-1^-^ and TIM-3^-^ phenotype avoids cytokine release syndrome (CRS) and neurotoxicity. The University of Texas MD Anderson Cancer Center spearheaded a phase I/IIa clinical study (NCT03056339) to assess the safety and efficacy of cord blood-derived cluster of differentiation 19 (CD19)-targeted CAR-NK cells (designated CAR19/IL-15 NK cells) in patients with relapsed or refractory CD19-positive B-cell hematological malignancies, such as non-Hodgkin lymphoma (NHL) and chronic lymphocytic leukemia (CLL). Results from this trial demonstrated a cumulative ORR of 48.6% across the cohort, with substantial variability by disease subtype: 100% ORR in low-grade NHL, 67% in CLL, and 41% in diffuse large B-cell lymphoma (DLBCL). The 1-year OS was 68%, with no graft-versus-host disease (GVHD) or neurotoxicity observed^[180]^. Critically, CAR-NKs persisted > 90 days post-infusion, confirming durable engraftment^[181]^. Targeting diverse antigens [e.g., EGFR deletion variant III (EGFRvIII), MHC class I polypeptide-related sequence A (MICA) and MHC class I polypeptide-related sequence B (MICB), natural killer group 2 member D (NKG2D) ligands] enhances tumor specificity while leveraging “off-the-shelf” availability^[182]^.

STING agonists: activating innate immune surveillance Stimulator of interferon genes (STING) agonists reprogram immunosuppressive TMEs by binding cyclic guanosine monophosphate-adenosine monophosphate (cGAMP), triggering TANK binding kinase 1 (TBK1)/interferon regulatory factor 3 (IRF3) phosphorylation and type I interferon production. This signaling cascade facilitates the maturation of DCs and enhances the infiltration of CD8^+^ T cells into the TME. In the context of HNSCC, a phase II clinical trial (NCT03937141) investigated the combination of ADU-S100 and pembrolizumab in adult patients with PD-L1-positive R/M HNSCC. The findings indicated that the ADU-S100 plus pembrolizumab regimen exhibited favorable tolerability and successfully induced abscopal effects^[183]^. Novel delivery systems - including pH-sensitive nanoparticles targeting TAMs and sustained-release intratumoral implants (e.g., MK-2118) - optimize bioavailability and reduce systemic toxicity^[1,184]^.

Epigenetic modulators: resetting immune evasion mechanisms Histone deacetylase inhibitors (HDACi; e.g., entinostat) and enhancer of zeste homolog 2 (EZH2) inhibitors (EZH2i; e.g., tazemetostat) reverse epigenetic silencing of immunogenic pathways. HDACi downregulate Treg-suppressive genes [*FOXP3*, cytotoxic T-lymphocyte-associated protein 4 (*CTLA4*)], while EZH2i reactivate endogenous retroviruses, inducing “viral mimicry” via dsRNA sensing pathways. The ongoing phase II ENCORE601 trial (NCT02437136) evaluates entinostat plus pembrolizumab in multiple tumors. In 76 treated NSCLC patients, the ORR was 9.2% (below the pre-specified threshold), median PFS was 2.8 months (95%CI 1.5-4.1), and median OS was 11.7 months (95%CI 7.6-13.4), with no new toxicities (including irAEs) observed. However, the combination missed its primary efficacy endpoint^[185]^. Demethylation of the interferon gamma (IFNG) promoter in circulating T cells served as a predictive biomarker for therapeutic response^[186]^. These agents also enhance chimeric antigen receptor T-cell (CAR-T)/NK cell function by reducing exhaustion and promoting memory phenotypes^[187]^.

In conclusion, a growing body of clinical and preclinical evidence indicates that synergistic treatment modalities can induce potent and durable anti-tumor responses in R/M HNSCC. This approach provides a potential pathway to achieving long-term disease control in historically intractable HNSCC subsets. Future research must focus on optimizing these combinations to maximize efficacy while diligently managing the associated toxicities.

## CONCLUSION

Resistance to immunotherapy in HNSCC is the result of complex, intertwined mechanisms involving both intrinsic tumor characteristics and extrinsic regulation by the TME. Future research should focus on the following directions: (1) developing multi-omics technologies to analyze the dynamic evolution of resistance; (2) exploring combination therapeutic strategies targeting both tumor cells and the microenvironment; (3) achieving precise classification based on biomarkers such as TMB and the spatial distribution of PD-L1; (4) developing efficient and low-toxicity immunotherapy combination models. Additionally, novel immunotherapies, such as CAR-T therapy and bispecific antibodies, hold promise for overcoming current therapeutic limitations. Through interdisciplinary collaboration and translational research, the ultimate goal is to comprehensively overcome immunotherapy resistance in HNSCC.

## DECLARATIONS

### Copyright

© The Author(s) 2025.
